# Active charge state control of single NV centres in diamond by in-plane Al-Schottky junctions

**DOI:** 10.1038/srep12160

**Published:** 2015-07-16

**Authors:** C. Schreyvogel, V. Polyakov, R. Wunderlich, J. Meijer, C. E. Nebel

**Affiliations:** 1 Fraunhofer-Institute for Applied Solid State Physics (IAF), 79108 Freiburg, Germany; 2 Department of Physics and Geoscience, University of Leipzig, 04103 Leipzig, Germany

## Abstract

In this paper, we demonstrate an active control of the charge state of a single nitrogen-vacancy (NV) centre by using in-plane Schottky-diode geometries with aluminium on hydrogen-terminated diamond surface. A switching between NV^+^, NV^0^ and NV^−^ can be performed with the Al-gates which apply electric fields in the hole depletion region of the Schottky junction that induces a band bending modulation, thereby shifting the Fermi-level over NV charge transition levels. We simulated the in-plane band structure of the Schottky junction with the Software ATLAS by solving the drift-diffusion model and the Poisson-equation self-consistently. We simulated the IV-characteristics, calculated the width of the hole depletion region, the position of the Fermi-level intersection with the NV charge transition levels for different reverse bias voltages applied on the Al-gate. We can show that the field-induced band bending modulation in the depletion region causes a shifting of the Fermi-level over NV charge transition levels in such a way that the charge state of a single NV centre and thus its electrical and optical properties is tuned. In addition, the NV centre should be approx. 1–2 μm away from the Al-edge in order to be switched with moderate bias voltages.

The nitrogen-vacancy (NV) centre shows well-known optical and electronic properties as a function of its charge state (NV^−^, NV^0^ and NV^+^). For electron-spin related applications in quantum physics, the NV^−^ centre shows very promising properties such as perfect photostability^1^, an optical initialization and read-out of its ground state electron spin^2^ and the optically detected magnetic resonance^3^ at ambient conditions which can be exploited for applications such as single-spin magnetometry^4^ and imaging in life science^5^. In addition, it fulfils all DiVincenzo-criteria (well-defined qubits, initialization to a pure state, universal set of quantum gates, qubit specific measurement and long coherence time) for a quantum computing application^6^ even at room temperature.

For most applications in quantum physics, it is essential to use NV^−^ centres as close as possible to the diamond surface (1–10 nm below the surface), in order to efficiently couple the emitted photoluminescence to a photonic waveguide or a microcavity for example^7^,^8^. However, the charge state of near-surface NV centres is strongly affected by surface defects, surface terminations and adsorbates and switches in an uncontrolled way between NV^−^, NV^0^ and presumably NV^+^ 9,10,11. Therefore, a control or stabilization of the charge state and the optical properties of the NV centre is very important and can be achieved by controlling the position of the Fermi level within the bandgap. This can be performed passively by the chemical control of the charge state via surface termination with oxygen^9^,^10^,^11^ or fluorine^12^ and actively by an electrical control^13^,^14^,^15^,^16^.

Addressing NV centres individually is necessary in order to operate single sensors or to entangle a large number of NV centres. It was recently shown that a swapping of the electronic spin to a nuclei leads to a very large T_2_ times^17^ (required for quantum information storage) as well as the entanglement could be conserved with high fidelity^18^. The main feature is based upon the electrical switching of single NV centres individually from NV^+^ (dark state, no electron spin) to NV^−^. This yields to new scheme of a full scalable quantum computer.

In this work, we demonstrate an active control of the charge state of a single NV centre by using a two-dimensional Schottky junction of aluminium on a hydrogen terminated diamond surface^19^,^20^. Aluminium forms a Schottky contact on H-terminated diamond surface inducing a lateral hole depletion layer next to the Al contact. By applying external potentials to the Al contact the band bending in the depletion region can be modulated, thereby shifting the Fermi level over NV charge transition levels to switch the NV charge state. We use confocal microscopy to detect the NV-photoluminescence in the depletion region of the Schottky junction. Since the NV^+^ centre does not show fluorescence upon optical excitation, whereas NV^0^ shows a zero phonon line at 575 nm and NV^−^ at 637 nm with their respective phonon side bands^9^ the different optical behaviour can be used for the charge state identification simply by spectrum analysis. We can show that by applying reverse bias voltages to the diode, we are able to switch from the non-fluorescent NV^+^ state to the fluorescent state NV^0^ and NV^−^ respectively. Upon switching off the diode, we are able to switch back to the non-fluorescent NV^+^ state.

## Experimental details

A Ib (001) 3 × 3 × 0.5 mm^3^ diamond plate from Element Six Ltd. was used as a substrate and a 300 μm thick, intrinsic and nitrogen-free epi-layer was grown homoepitaxially onto this substrate using an ellipsoidal shaped microwave plasma-enhanced chemical vapour deposition (MWPECVD) reactor^21^.

After growth, the substrate was removed by laser cutting, the epi-layer was polished using chemical-mechanical polishing^22^ to get an atomic smooth surface and then cleaned wet chemically (H_2_ SO_4_+HNO_3_ with a ratio of 3:1) resulting in oxygen-termination of the surface.

For micro-photoluminescence (μPL) characterisations, we used a commercial confocal microscope (LabRam BX41, Horiba Jobin Yvon) with a laser excitation wavelength of 532 nm and an intensity of about 1 mW. The photoluminescence signal collected by the microscope objective was then routed through a fibre to a monochromator and the spectrum was measured with a CCD-Camera (Synapse, Horiba Jobin Yvon). For photoluminescence-intensity mappings, the integrated intensity in the whole spectral range from 570 nm to 900 nm is recorded with a photomultiplier tube (RS943-02, Hamamatsu). Please note that all the presented spectra in this paper were acquired with the same excitation and detection conditions. Each spectrum was normalized to the amplitude of the first order Raman line to allow comparison and evaluation.

**Figure 1:**
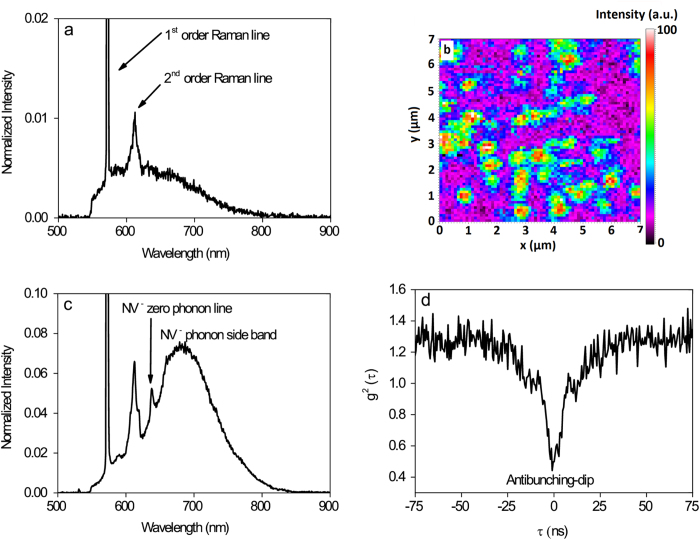
Formation of NV centres. **(a)** Photoluminescence spectrum of the grown diamond epi-layer with oxidized surface, normalized to the first order Raman line. The first and second order Raman lines as well as a weak background signal are visible. **(b)** A PL-intensity mapping performed on the diamond surface shows clear bright spots indicating NV-photoluminescence. **(c)** An NV^−^ centre photoluminescence spectrum measured on one of the bright spots in the mapping image in (**b**). **(d)** The corresponding second-order photon autocorrelation measurement performed with a Hanbury-Brown and Twiss Interferometer setup, which shows a clear antibunching dip which indicates single photon emission.

The confocal μPL spectrum of the grown, polished and oxidized diamond epi-layer (Fig. 1a) shows the first order (at 572 nm) and second order (at 612 nm) Raman lines and a background spectrum. This background is from the surface as it vanishes when spectra were taken from deeper in the bulk. The origin of this background is unclear at the moment and requires further investigation.

In the next step, the whole diamond sample was implanted with nitrogen ions (^15^N) with a fluence of 10^8^ ions/cm^2^ and energy of 5 keV. The resulting implantation profile, simulated with the software SRIM (Stopping and Range of Ions in Matter)^23^, shows a Gaussian density distribution with a centre position of 8.1 nm from the surface and a standard deviation of 3.1 nm. In order to activate the diffusion of carbon-vacancies (which are also created during implantation process), the sample was annealed in vacuum for 2 h at 800 °C. This results in NV centre formation since the carbon-vacancies are trapped by nearby substitutional nitrogen atoms. A PL-intensity mapping performed on the diamond surface shows bright spots (Fig. 1b). By measuring the spectrum (Fig. 1c) and the second-order photon autocorrelation with a Hanbury-Brown and Twiss Interferometer setup^1^,^24^,^25^, which shows a clear antibunching dip at around *τ* = 0 s (Fig. 1d), the bright spots could be identified as single NV^−^ centres. An analysing of PL-mapping images from different regions of the diamond surface (mapping size of 20 × 20 μm^2^ each) reveals an average NV density of approximately 1 × 10^7^ cm^−2^. This would correspond to a formation efficiency of approximately 10% which is in agreement with formation efficiency values reported in literature^26^,^27^.

For the realisation of an in-plane Schottky diode, the surface was first hydrogen-terminated (H-terminated) by applying a pure hydrogen plasma in the MWPECVD-reactor which results in the formation of a two-dimensional hole accumulation layer at the surface^19^. The NV emission is quenched by the interaction with the two-dimensional hole accumulation layer at the diamond surface, i.e. the NV centres were switched to the non-fluorescent state NV^+^. This was proved by a PL-intensity mapping which shows no bright, single spots at the surface.

**Figure 2:**
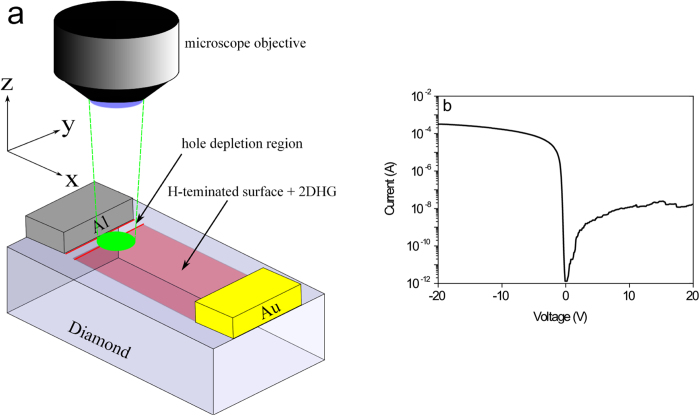
In-plane Schottky diode from diamond. **(a)** Schematic figure of an in-plane Al Schottky diode on H-terminated diamond surface. A confocal μPL measurement was performed at the edge of the Al contact (green spot) to investigate the bias-voltage dependent variation of NV emission in the depletion region. **(b)** The current-voltage properties of the fabricated in-plane Al-Schottky diode measured at ambient conditions at 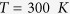.

Then, aluminium (Al) and gold (Au) contacts with a thickness of 200 nm each were deposited on the diamond surface using photolithography in combination with thermal evaporation of the metals. Al is used as a Schottky contact showing a barrier height of about 570 meV and Au is used as an Ohmic contact^19^. The contacts exhibited a dimension of 1 mm × 300 μm and were separated from each other by 400 μm. The in-plane diamond Schottky diode is shown schematically in Fig. 2a. The current-voltage characteristic of the fabricated in-plane Schottky diode, measured at ambient conditions, is shown in Fig. 2b. An on/off ratio of about 5 orders of magnitude is achieved.

To investigate the influence of external bias voltages applied to the Al-gate on the NV emission in the depletion region at the edge of the Al contact (depicted in Fig. 2a), we used an objective with a long working distance (Olympus SLMPLN, 100x, NA = 0.6, WD = 7.6 mm) to avoid short circuits between the microscope objective and the metallized and wired diamond surface.

## Results

**Figure 3:**
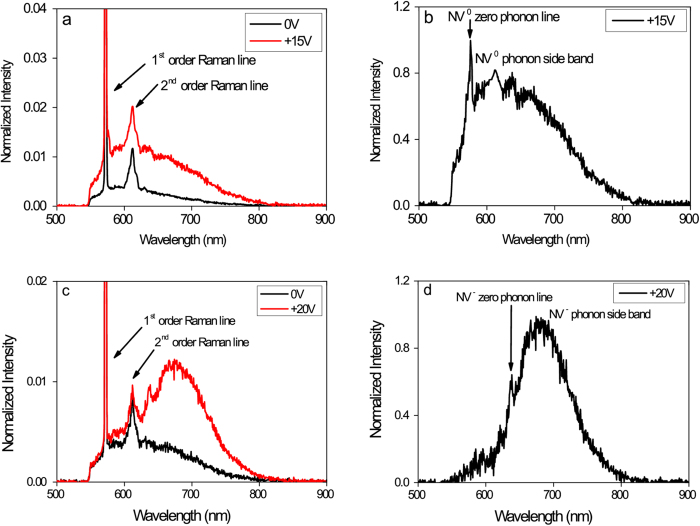
Tuned optical properties of a single NV centre. **(a)** For zero-potential condition there is only a PL-background of the diamond surface (black spectrum) since the NV centre is in the non-fluorescent NV^+^ state due to the interaction with the two-dimensional hole accumulation layer. Upon applying a reverse bias potential of +15 V we detect a spectrum of NV^0^ emission (red spectrum). **(b)** The background corrected spectrum of the NV^0^ emission displayed in graph (**a**). The zero phonon line at 575 nm and the respective phonon side band is visible. **(c)** Upon applying a reverse bias potential of +20 V we detect a spectrum of NV^−^ emission (red spectrum). **(d)** The background corrected spectrum of the NV^−^ emission displayed in graph (**c**). The zero phonon line at 637 nm and the respective phonon side band is visible. Each spectrum in Figs a–d was normalized to the amplitude of the first order Raman line to allow comparison and evaluation.

For zero-potential conditions there is only a PL-background of the diamond surface (black spectrum in Fig. 3a), since the NV centre is in the NV^+^ state which shows no fluorescence as mentioned above. Upon applying a reverse bias voltage of +15 V, the measured spectrum shows NV^0^ emission with the characteristic zero phonon line at 575 nm and its phonon side band (red spectrum in Fig. 3a). By subtracting the background (represented by the black spectrum in Fig. 3a) we can see a clear signature of NV^0^ emission (Fig. 3b). By increasing the reverse bias voltage to +20 V, the measured spectrum shows NV- emission with the characteristic zero phonon line at 637 nm and its phonon side band (red spectrum in Fig. 3c). By subtracting the background (represented by the black spectrum in Fig. 3c) we can see a clear signature of NV^−^ emission (Fig. 3d).

In addition to that, a second-order photon autocorrelation measurement was performed on the same spot with the above mentioned reverse bias conditions. For both NV^0^ and NV^−^ emission, the result shows an antibunching dip, each with a minimum value slightly above 0.5 (see Fig. 1d). For being a single photon emitter, the minimum value should be ideally below the value of 0.5^1^,^24^,^25^. But since the PL-background of the surface is large and can thus not be neglected, the clear signature of the antibunching dip is minimized. For this reason, we interpret the measured antibunching dip nevertheless as a proof for single photon emission, i.e. being a single NV centre.

After switching off the applied bias voltage (back to zero-potential condition), the recorded PL-spectrum revealed only the background of the diamond surface as shown in the black spectrum of Fig. 3a,c, which corresponds to the non-fluorescent NV^+^ state. This switching back and forth of single NV centres from NV^+^ to NV^0^ and NV^−^ and thus the tuning of its optical and electrical properties was reproducible. The required reverse bias potential to be applied to the Al-contact for switching the centre from NV^+^ to NV^0^ and NV^−^ is different for each centre. The reason for this will be discussed later.

**Figure 4:**
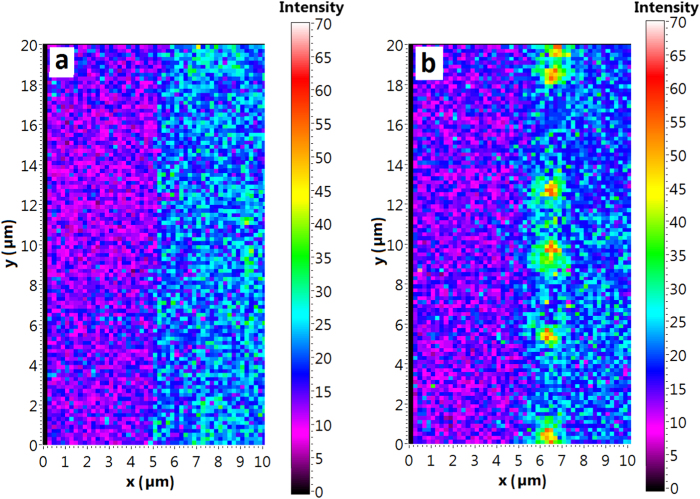
PL-Intensity mapping in the depletion region. A PL-intensity mapping performed on the diamond surface at the edge of the Al-Schottky-contact (rectangle area on the left with lower PL intensity). Compared to the zero-potential condition **(a)** there are bright spots visible at the edge of the Al-contact **(b)** upon applying a reverse bias potential of +20 V indicating NV^−^ photoluminescence in the hole depletion region of the Schottky-junction.

Since the Fermi level in the area outside the depletion region is not altered by the bias voltage, it is not possible to switch non-fluorescent NV^+^ centres to NV^0^ and NV^−^ upon applying reverse bias voltages. This was confirmed by the result of a PL-intensity mapping performed at the edge of the Al-contact. For the zero-potential condition there is just an intensity background visible of the diamond surface next to the Al-contact (Fig. 4a) whereas for the reverse bias condition from +15 V to +20 V (Fig. 4b), only at the edge of the Al-contact there are bright spots which are referred to NV luminescence.

We also performed spectral measurements under forward bias conditions but we could neither detect any change in the spectra (i.e. only the background spectrum was observed) nor observed electroluminescence from NV centres.

## Discussion

**Figure 5:**
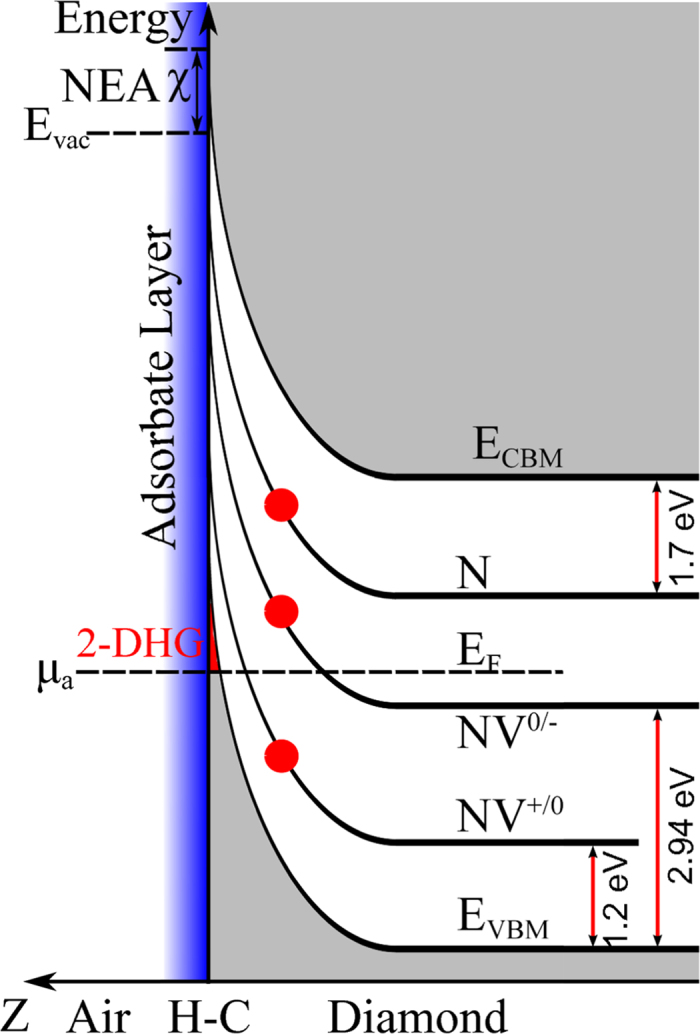
H-terminated diamond surface and NV charge transition levels. Schematic energy band diagram of an H-terminated diamond from the surface into the bulk (z-axis as depicted in Fig. 2a). E_vac_ is the vacuum level, χ is the negative electron affinity, E_VBM_ and E_CBM_ is the valence band maximum and the conduction band minimum respectively, E_F_ is the Fermi level. H-C indicates the carbon-hydrogen bond at the surface and μ_a_ is the chemical potential of the aqueous wetting layer covering the diamond surface. 2-DHG is the two-dimensional p-type channel. Also shown are the charge transition levels NV^+/0^ (1.2 eV above valence band maximum E_VBM_) and NV^0/−^ (2.94 eV above valence band maximum E_VBM_) as well as the nitrogen donor level N (1.7 eV below conduction band minimum E_CBM_). The red circles indicate schematically the position of single nitrogen atoms and NV centres below the surface generated by implantation (centre position is 8.2 nnm below the surface with a standard deviation of 3.4 nm).

To understand the formation of the in-plane Schottky-junction and the bias-dependent NV emission, we have first to consider the band bending at the surface of an H-terminated diamond which is shown schematically in Fig. 5 and will be explained in the following. Please note that this schematic band diagram is from the surface into the bulk of diamond, i.e. it is in z-direction as depicted in Fig. 2a.

Hydrogen terminated diamond shows a negative electron affinity χ, such that the chemical potential μ_a_ of the aqueous wetting layer covering the diamond surface is below the valence band maximum (VBM) giving rise to tunnelling of electrons from the valence band into the lowest unoccupied electronic states of the aqueous wetting layer (transfer doping model)^19^,^28^,^29^. This induces a band bending from the surface into the bulk and the formation of a hole accumulation layer at the diamond surface^19^. The hole sheet density is determined by the quality of the H-termination and of the diamond surface itself. Typical values for the hole sheet density vary between 10^11^ and 10^12^ cm^−2^ 19. Calculations by Nebel *et al.*^19^ show that this hole accumulation layer extends approximately 1–4 nm into the diamond bulk. Since the de Broglie wavelength of the holes is larger than the depth of this hole accumulation layer, the channel has a two-dimensional character.

The charge transition level of a defect centre is defined as a level at which the centre takes up or loses an electron when the Fermi level crosses this level. Towards the surface, the NV charge transition levels NV^+/0^ (1.2 eV above VBM^30^) and NV^0/−^ (2.94 eV above VBM^31^) are moving above the Fermi level as schematically shown in Fig. 5. As both NV charge transition levels are unoccupied near the surface, the NV photoluminescence is quenched. In our experiment the depth of the NV layer is approximately 8 nm, i.e. it is spatially separated from the hole accumulation layer which is also shown schematically in Fig. 5 (green area). The quenching of NV centres can be explained by tunnelling transitions from the nearby hole channel and by the fact that the typical hole sheet density of approximately 10^12^ cm^−2^ exceeds the sheet density of implanted nitrogen ions four orders of magnitude.

**Figure 6:**
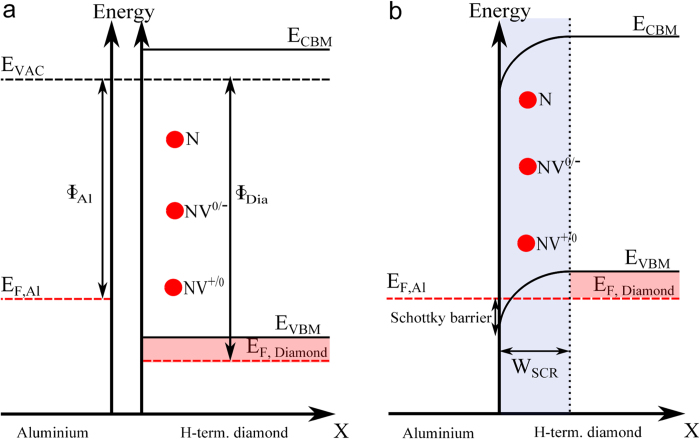
Formation of in-plane Schottky junction. **(a)** Schematic lateral band diagrams of the Al and H-terminated diamond surface, i.e. Al is not in contact with the H-terminated diamond surface (x-axis as depicted in Fig. 2a). Φ_Al_ and Φ_Dia_ is the work function of Al and H-terminated diamond surface respectively. The charge transition levels NV^+/0^ and NV^0/−^ as well as the nitrogen doping centre (P1-centre) are also displayed (red circles). **(b)** Schematic lateral band diagram of the in-plane Al-Schottky junction, i.e. Al is in contact with the H-terminated diamond surface (x-axis as depicted in Fig. 2a). A Schottky barrier for holes and a hole depletion region at the edge of Al contact is induced. In this case the Fermi-level is below NV^+/0^ and NV^0/−^ charge transition level, thus the single NV centre is in the NV^+^ state.

To explain the formation of an in-plane Schottky junction between an Al-contact and the H-terminated diamond surface, we have to consider their band structure which is shown schematically in Fig. 6a,b. Please note that these band diagrams are now parallel to the diamond surface, i.e. in x-direction as depicted in Fig. 2a.

Directly below the H-terminated diamond surface, the Fermi-level is below the valence band maximum which means that the work function Φ_Dia_ is larger than the work function Φ_Al_ of aluminium (Fig. 6a). As a consequence, a Schottky barrier for holes and a hole depletion region in the two-dimensional p-type surface conductive layer is induced at the edge of the Al contact^19^ (Fig. 6b) when Al is in contact with the H-terminated diamond surface. The NV charge transition levels NV^+/0^ and NV^0/−^ are also displayed in the lateral band diagrams in Fig. 6a,b. For our Schottky diode, NV centres are near the Al contact covering the whole diamond surface, following the band bending of the Schottky contact, i.e. the distance of each single NV centre from the Al-edge is different.

### Simulation of the planar Schottky junction

For the explanation of bias-dependent NV emission (Fig. 3a–d), the effect of band bending modulation in the hole depletion region upon applying bias potentials on Al-Schottky contact has to be considered. For this purpose and to get a deeper insight into the operation of the planar diamond Schottky diode, we carried out simulations using the 2D device simulator ATLAS from Silvaco^32^. To simulate different reverse bias conditions of the diode, the current continuity equations based on the drift-diffusion model (calculation of charge carrier distribution) are self-consistently solved with the Poisson equation (calculation of potential distribution). The continuity equations describe the way the electron and hole densities evolve as a result of generation, transport and recombination processes. The mathematical models used in the ATLAS software are described in the user’s manual of that software^32^.

**Figure 7:**
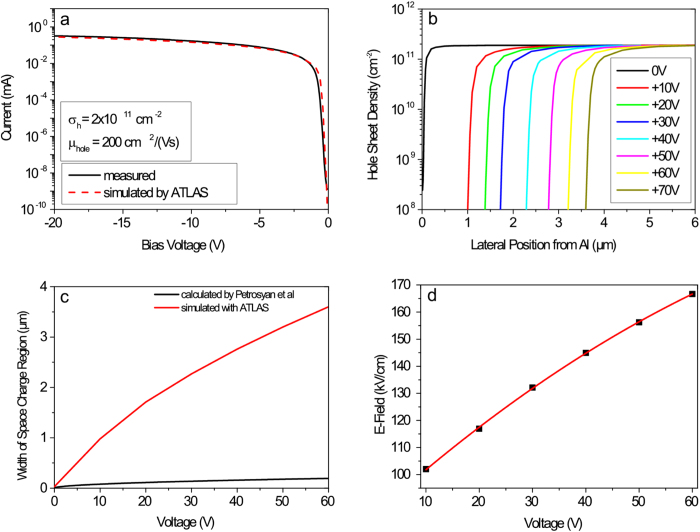
Simulation of the planar Schottky junction. **(a)** Simulated current-voltage characteristic of the planar Schottky junction for a hole sheet density of 2 × 10^12^ cm^−2^ which shows a good agreement with the measurement. **(b)** Calculated hole hole sheet density for different lateral positions from the Al-contact edge (position: 0 μm) at different reverse bias potentials. **(c)** Increase of the space charge region as a function of reverse bias potential, calculated with the software ATLAS and deduced from Fig. b) as described in the text and a comparison with the calculation by Petrosyan *et al.*^33^ The red curve can be described by the function 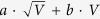 (with 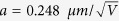 and 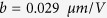). **(d)** Calculated values for the electric field inducing a band bending modulation in the space charge region.

In the first step, we calculated the electrical properties of the planar Schottky diode, i.e. the current-voltage characteristic. For this, we placed a negative counter charge with a defined sheet density σ_e_ at the air/diamond interface (i.e. in principle in the atmospheric adsorbate layer on the surface of H-terminated diamond) to generate a hole accumulation layer with approximately the same sheet density σ_h_ below the surface. The value of this counter charge σ_e_ and thus the hole sheet density σ_h_ was adjusted to get the best agreement of the simulated with the measured current-voltage characteristics under forward bias conditions (Fig. 7a). For this case, we have a hole sheet density of approximately 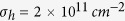 and a hole mobility of 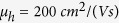
which are in agreement with typical values measured and reported in literature^19^.

In the next step, we calculated the hole sheet density in the two-dimensional hole channel as a function of the lateral position from the Al-Schottky contact for different reverse bias potentials (Fig. 7b). From this calculation, we can deduce the width of the hole depletion region at the edge of the Al-Schottky contact for different reverse bias potential conditions. For voltages above +10 V, it is in the order of some micrometres. Thus, an NV centre being within the range of 1–2 μm from the Al-edge can be switched from NV^+^ to NV^−^ and vice versa with moderate values of the applied bias potential. This result is in accordance with the experimental results shown in the intensity mapping image in Fig. 4b, where NV centres are approximately 1–2 μm away from the Al-contact.

Furthermore, we compared our calculated widths with those calculated by Petrosyan and Shik^33^ as well as by Gelmont *et al.*^34^ (Fig. 7c). They calculated the general features of a junction between a two-dimensional electron gas and a metal contact with Schottky properties which is also valid for a two-dimensional hole gas. The depletion region width is expressed as follows: 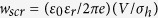 where ε_0_ is the dielectric constant, ε_r_ the relative dielectric constant of diamond, V the applied potential on Al-Schottky contact and σ_h_ the hole sheet density. For this calculation, they assumed a delta-function-like thickness of the hole channel, i.e. it has effectively no (finite) thickness. They mentioned, that in case of a three-dimensional hole channel, the hole depletion region width depends on the square-root of the applied bias potential, i.e. 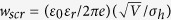. For our real and simulated device the hole channel has a finite thickness, we would thus expect a superposition of linear and square-root dependence of the hole depletion region width on the applied potential, which was approved by fitting a function 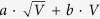 to the simulated data (with fitting parameters 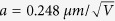 and 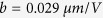).

But our calculated widths are considerably larger than those calculated with the formula used by Gelmont *et al.*^34^. The difference can be deduced from the fact that in our simulation, the quantization effects of the two-dimensional hole gas were neglected, which results in a different hole distribution from the surface into the bulk of diamond. In addition, the surface band bending of a H-terminated diamond surface as well as the Schottky barrier height of a defined value was not considered by Petrosyan and Gelmont^33^,^34^. Nevertheless, it can be stated that the depletion region between the 2D hole channel and the Al-Schottky contact is much larger than the thickness of the channel itself, which is approximately 1–4 nm^19^.

As the potential drop takes place within the space charge region of defined width, we can calculate the electric field inducing a band bending modulation in that region (Fig. 7d). For voltages up to +60 V, the electric field is approximately 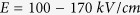, i.e. it is roughly two orders of magnitude below the breakdown voltage of diamond of 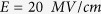
35.

**Figure 8:**
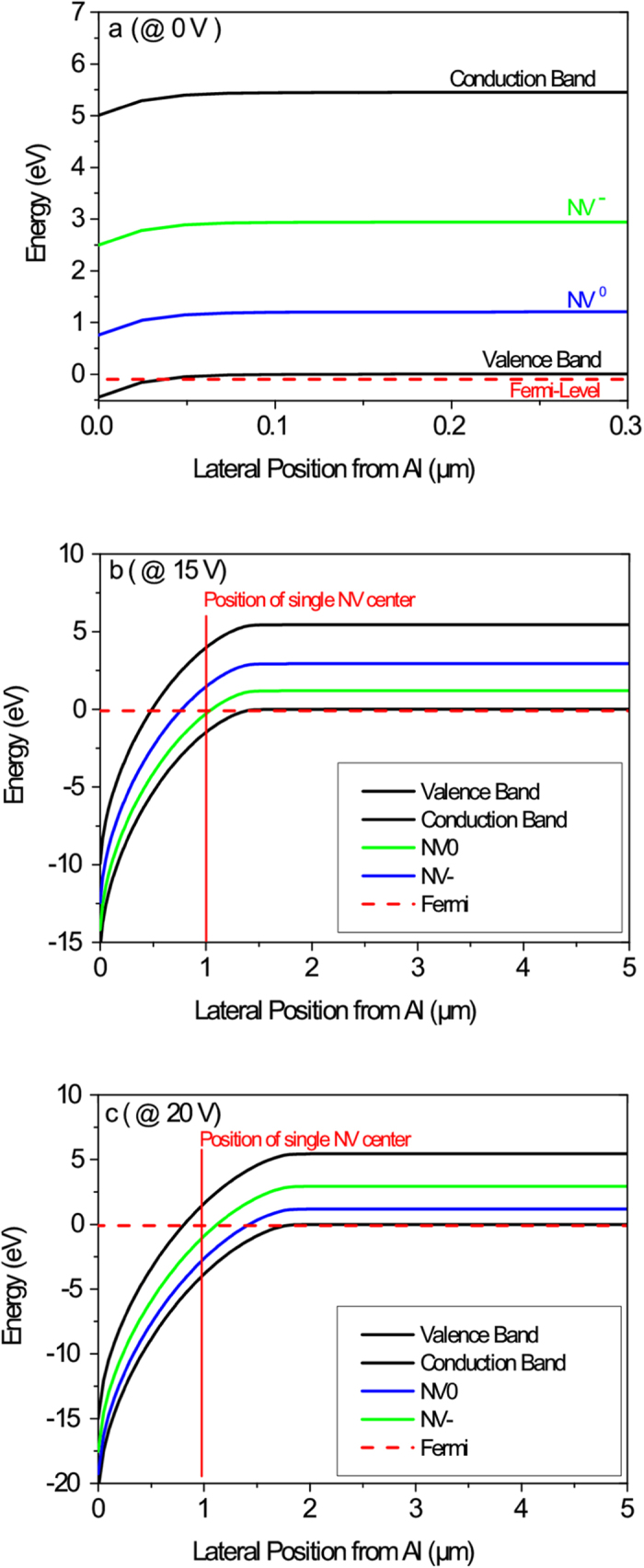
Simulation of band bending modulation in the depletion region. With the software ATLAS simulated band structure in the depletion region for **(a)** zero-potential condition, **(b)** reverse bias potentials of +15 V and **(c)** +20 V. The NV charge transition levels (blue and green lines) are also displayed as well as the Fermi-level (red dotted line) which is assumed to be approximately 90 meV^19^ below the valence band maximum outside the depletion region. Furthermore, as a representative case, we set the position of the single NV centre 1 μm from the Al-contact edge (vertical red line) to demonstrate the different charge states for different potential conditions of the diode.

The bias-dependent NV emission can be understood by simulating the band bending modulation and the intersection of the Fermi-level with NV charge transition levels for different reverse bias potentials (0 V, +15 V and +20 V). The results are displayed in Fig. 8a–c. For discussing the results, we consider now a specific case, i.e. we assume the distance of an NV centre from the Al edge to be 1 μm and the position of the Fermi-level outside the hole depletion region to be approximately 90 meV 19 below valence band maximum.

For zero-potential condition, the Fermi-level is below the NV^+/0^ level, which means the NV centre is in the non-fluorescent state NV^+^ (Fig. 8a). Upon applying a reverse bias potential on Al-contact, the Fermi-level first crosses the NV^+/0^ (at +15 V, Fig. 8b) and then the NV^0/−^ (at +20 V, Fig. 8c) charge transition level, i.e. the NV centre is switched from NV^+^ to NV^0^ and then to NV^−^. This result agrees very well with our experimental results.

To generalize these findings, we can state that by increasing the reverse bias voltage, the band bending in the hole depletion region is modulated in such a way that the Fermi level first crosses the NV^+/0^ and then, at higher reverse bias potential, the NV^0/−^ transition level. Depending on the relative position of a single NV centre with respect to the Al edge different bias potentials are required to achieve a charge state switching. The larger the distance of an NV centre from the Al edge the higher reverse bias potentials have to be applied to switch the charge state from NV^+^ to NV^0^ and NV^−^. Since the lateral resolution of the long working distance objective of the confocal microscope is in the order of 800 nm and the Al edge is not perfectly sharp (i.e. it shows a falling slope), it is difficult to prove the above mentioned correlation between NV centre distance from the Al-edge and the required reverse bias potential for charge state switching.

Furthermore it is obvious that under forward bias conditions we cannot expect a switching of an NV centre since the Fermi-level (which is for most NVs already below the NV^+/0^ level at 0 V) is pushed further down towards the valence band.

Such two-dimensional properties which we have simulated with the software ATLAS can be expected only theoretically and ideally, as the real surface is affected by several aspects such as a) surface and bulk defects which may pin the surface Fermi level, b) surface roughness and c) as well as Ions in the Helmholtz layer of the adsorbate Film in close vicinity to the hole channel.

In order to understand the underlying mechanisms for charging and discharging NV centres which results in the tuning of its optical properties, we are currently performing measurements of the time constant for dynamic switching NV centres upon switching the diode at different conditions (laser light intensity, temperature, etc.). We assume that holes play a dominant role in the observed effects due to the 2D-hole channel at the H-terminated diamond surface. In other words, a charging and discharging of NV centres is induced by capture and release of holes, depending on the bias condition.

### Summary and Conclusion

In the depletion region of a planar Al-Schottky diode from diamond, we are able to switch the charge state of a single NV centre from NV^+^ to NV^0^ and NV^−^ (by increasing the reverse bias potential) and vice versa (by decreasing the reverse bias potential towards zero-potential condition).

We also performed simulations of the electrical properties of the planar Schottky diode using the Software ATLAS by solving the drift-diffusion model self-consistently with the Poisson equation. The results show a good agreement of the simulated current-voltage characteristic with the experimental results. And we could show that the width of the space charge region is in the order of 1–2 μm for voltages up to +20 V. Furthermore, we could show that the field-induced band bending modulation in the depletion region causes a shifting of the Fermi-level over the NV charge transition level, thereby switching the charge state of a single NV centre and thus the tuning of its electrical and optical properties.

To elucidate the mechanism and dynamics for switching the charge state of NV centres, measurements of the time constant for charging and discharging NV centres at different conditions (temperature, laser intensity, etc.) are required. And we are currently introducing a detailed theoretical discussion of the defect properties based on ab-initio calculations to understand the charge state switching mechanisms which will be presented in an up-coming paper.

Using this planar Schottky-diode thus enables us the stabilization of the NV^−^ state, so that the extraordinary optical and spin properties can be used for many quantum physical applications. In addition to that, by switching between NV^−^ and NV^+^ and vice versa, it opens the door to quantum computing applications. In a quantum computer, the electron spin of the NV^−^ centre can be used for fast information processing and the spin of a nucleus for (long timescale) information storage. For processing, storage and optical manipulation and read-out of quantum information, a switching between NV^−^ and NV^+^ is required. The Al-gates could be structured in such a way that each NV centre can be addressed individually. This yields to new scheme of a full scalable quantum computer.

## Methods

### Fabrication of in-plane Schottky diode from diamond with NV centres

For realizing a two-dimensional Schottky-diode we prepared a diamond sample. An Ib (001) 3 × 3 × 0.5 mm^3^ diamond plate from Element Six Ltd. was used as substrate and a 300 μm thick intrinsic, nitrogen free epi-layer was grown homoepitaxially onto this substrate using the ellipsoidal shaped MWPECVD (Microwave Plasma-enhanced Chemical Vapour Deposition) reactor^21^. The process parameters used were 210 mbar gas pressure at a gas flow of 290 sccm H_2_ and 10 sccm CH_4_ (which corresponds to a CH_4_/H_2_ ratio of 3.5%) and a microwave power of 2.1 kW. Under these conditions, the measured growth temperature was about 800 °C. The growth rate was 1.7 μm/h.

A shallow nitrogen-ion beam implantation was performed by J. Meijer *et al.* The implantation energy was 5 keV and the sheet density 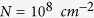. The resulting implantation profile shows a Gaussian density distribution with a centre position of 8.1 nm and a standard deviation of 3.1 nm that has been simulated with the software SRIM (stopping and range of ions in matter)^23^. The formation of NV centres was achieved by annealing the sample for 1 h at 800 °C.

Hydrogen termination was achieved by applying pure hydrogen plasma in the MWPECVD reactor at a pressure of 100 mbar, with a microwave power of 1.9 kW. The duration of the termination process was 2 minute at a temperature of around 500 °C.

The fabrication of the in-plane Schottky diode was realized by using photolithography in combination with thermal evaporation of Al and Au with a thickness of 200 nm each on the diamond surface. The contacts were 1 mm × 300 μm large and separated from each other by a distance of 400 μm.

### Experimental setup for optical detection

Our confocal microscope set-up is equipped with a diode-pumped and frequency-doubled Nd:YAG-laser operating at a wavelength of 532 nm with an output power of 5 mW and a commercial confocal microscope (LabRam BX41, Horiba Jobin Yvon). The laser beam is passed to the microscope through an optical fibre and focused onto the sample by the microscope objective. The laser power illuminating the sample is 1 mW due to losses in the optical path of the microscope setup. The resulting photoluminescence signal is collected by the same objective and the excitation wavelength is spectrally filtered out using notch filter. The light beam is subsequently focused onto a pinhole for spatial filtering and routed via a fibre to a monochromator (iHR-320, Horiba Jobin Yvon). As detector a Peltier-cooled CCD-Camera (SYNAPSE, Horiba Jobin Yvon) was used.

To investigate the tuned NV emission, we used an objective with a long working distance (Olympus LMPlanFLN, 50x, NA = 0.5, WD = 10.6 mm) to avoid a short circuit between the microscope objective and the metallized and wired diamond surface.

## Additional Information

**How to cite this article**: Schreyvogel, C. *et al.* Active charge state control of single NV centers in diamond by in-plane Al-Schottky junctions. *Sci. Rep.* **5**, 12160; doi: 10.1038/srep12160 (2015).
